# Precise Supervision of Enterprise Environmental Protection Behavior Based on Boolean Matrix Factorization under Low Carbon Background

**DOI:** 10.3390/ijerph19137739

**Published:** 2022-06-24

**Authors:** Wei Zhou, Feipeng Guo

**Affiliations:** 1 School of Management and E-Business, Zhejiang Gongshang University, Hangzhou 310018, China; 20020200028@pop.zjgsu.edu.cn; 2 Modern Business Research Center, Zhejiang Gongshang University, Hangzhou 310018, China

**Keywords:** low carbon, enterprise environmental protection behavior, Boolean matrix factorization, precise supervision, preventive measures

## Abstract

Supervising the environmental protection behavior of enterprises is a key strategy to achieve “carbon peaking and carbon neutrality”. This research innovatively proposes the concept of precise supervision, aiming to implement differentiated supervision measures for different types of enterprises, and realize the precise supervision method of enterprise environmental protection, which is different from the traditional supervision mode. Firstly, this paper proposes a novel MEBF+ method based on the benchmark algorithm MEBF, and obtains MEBF++ after incorporating the model bias. Secondly, based on the dataset of environmental supervision and certification of listed Chinese companies, the accuracy and robustness of the proposed method are verified by using multiple evaluation indicators. Finally, based on the analysis of the experimental results, two precise supervision concepts, narrow and broad, are proposed under the low-carbon background. The results show that compared with the benchmark method, the accuracy of the proposed method has been improved to a large extent. In addition, the precise supervision proposed in this paper can help reduce the consumption of manpower and resources as well as unite the public to monitor the environmental protection behavior of enterprises.

## 1. Introduction

With the goal of “carbon peaking and carbon neutrality” being put forward, the low-carbon behavior of enterprises is of great significance for the sustainable development of society and the economy [1]. The traditional supervision method is to treat all enterprises equally, or to randomly select enterprises for supervision, and it is difficult to achieve satisfactory results without establishing a corresponding supervision mechanism according to the characteristics of enterprises [2]. At the same time, blind supervision will seriously affect the efficiency of actual operation, resulting in the waste of manpower and resources, and it is difficult to regulate enterprise behavior [3,4]. In order to deal with the above problems, promote environmental governance, and facilitate the smooth implementation of carbon emission reduction, government departments have published many policies forcing enterprises to improve their behavior and improve the regulatory system [5,6]. For example, the Ministry of Ecology and Environment of China implemented the positive list system in March 2020 [7]. The core of the system is to implement classified supervision, reduce the on-site inspection of enterprises that meet the required standards in a stable manner, and increase the investigation and punishment of severely polluting enterprises.

In view of the improvement in the environmental protection behavior of enterprises, previous studies have verified that low-carbon behavior is beneficial to enterprise profits from various perspectives, ensuring the stable generation of economic value and environmental value, and contributing to the sustainability of development, etc. [8,9,10,11,12,13]. The focus is on advocating enterprises to take the initiative to improve their behavior, as well as suggestions on measures for departmental supervision [14,15,16,17]. However, due to the great differences in the status quo of enterprises [18], it is difficult for departments to make use of limited manpower and resources to carry out reasonable supervision, resulting in an imbalance between the actual supervision measures of the departments and the improvement of enterprise pollution behaviors, and the effectiveness of supervising the environmental protection behavior of enterprises is low [19]. Therefore, some scholars have carried out research from multiple perspectives and proposed relevant measures that are conducive to the improvement of supervision efficiency, so as to alleviate the current predicament with the unsatisfactory government-independent supervision model. On the one hand, the public generally maintains a positive attitude towards the implementation of departmental environmental protection policies, which helps to improve the efficiency of government supervision [20]. On the other hand, the role of public scrutiny in restricting enterprises’ illegal activities cannot be ignored. Under relatively reasonable supervision, enterprises are more motivated to comply with environmental protection policies [21]. In addition, the implementation of government supervision can use strong credit constraints to establish a coordinated supervision mechanism of appropriate strength [22]. At the same time, combining multi-party data to accurately assess enterprises can also improve the efficiency of supervision [23]. In addition, some scholars regard the balance between enterprise resources as a Boolean value, which is not only helpful to the implementation of departmental supervision, but also beneficial to the sustainable development of enterprises [24]. However, the above research on the environmental supervision of enterprises still pays more attention to the implementation suggestions of supervision and the handling of contradictions after supervision. There is little research on the choice of target before supervision and the choice of differential supervision mode of target enterprises. It is difficult to improve the efficiency of supervision and reduce the consumption of manpower and resources.

From the above, it can be seen that there are still some problems in the existing research, including: (1) Many scholars’ studies on low carbon are mostly about measures related to energy conservation and emission reduction, and there is a lack of targeted suggestions based on the current situation of enterprise environmental protection; (2) At present, the supervision of enterprises is still dominated by compulsory measures, without taking into account the huge differences in the actual situation of different types of enterprises; (3) Existing research has not yet integrated multi-domain knowledge to achieve the precise supervision of enterprise environmental protection behavior. Therefore, this paper proposes to analyze each enterprise before supervision, predict the probability of enterprises’ environmental protection indicators reaching the standard by using Boolean matrix factorization (BMF), and classify enterprises according to actual needs. Adopting specific solutions for different types of enterprises not only ensures the enhancement of supervision efficiency, but also ensures the stability of supervision. At the same time, this strategy provides relevant supervision authorities with a regulatory method that is more in line with the status quo of enterprises, saves manpower and resources, and contributes to the accelerated development of carbon emission reduction. Moreover, it is easier for the public to participate in the supervision game of enterprise environmental protection behavior, which further promotes the realization of “carbon peaking and carbon neutrality”.

In order to solve the above problems, this paper proposes an improved Median Expansion for Boolean Factorization method (MEBF), so as to realize the precise supervision of enterprise behavior by relevant departments. The contributions of this study are as follows:

1. This paper proposes to predict the probability of each enterprise’s environmental protection behavior compliance before supervision, and based on this, the precise supervision of each enterprise is realized;
2. An innovative improvement is achieved on the benchmark method MEBF. On the one hand, an improved stochastic gradient descent (SGD) method is proposed to replace the original optimization scheme. On the other hand, the model bias is introduced and integrated with the former, which further reduces the deviation degree after the algorithm iteration and improves the prediction accuracy;
3. Based on the empirical analysis of the experimental results, a precise supervision strategy is proposed, and targeted preventive measures are given, which provides a certain theoretical and practical basis for enterprises to achieve a low-carbon and environmentally friendly economy.

The main content of the remainder of this paper is as follows: In Section 2, the data source of this paper is explained, an improved method for the Boolean median extension factorization of the benchmark method is proposed, and the related evaluation indicators are introduced. In Section 3, the evaluation indicators are used to compare the accuracy of each algorithm in different data, and the robustness of the proposed method is verified. In Section 4, the algorithm results are discussed, the precise supervision mode is proposed, and the core processing steps of the model are briefly demonstrated. In Section 5, the main research conclusions of this paper are introduced, and corresponding policy suggestions are given, as well as future research directions.

## 2. Materials and Methods

### 2.1. Dataset Source

The data in this paper comes from the CSMAR database (China Stock Market and Accounting Research Database) [25]. By collecting multi-party data, making use of national public resources, and developing economic and financial fields according to China’s actual national conditions, the institution finally completes the creation of an accurate research-based database.

This paper uses the environmental supervision and certification disclosure tables of listed companies from 2017 to 2019, including 3467, 3565, and 3756 companies, respectively. A partial sample of the 2019 data is shown in Table 1, and the format of each year’s data is similar. It includes seven enterprise environmental protection behavior standards, including KPU (Key Pollution Unit), PES (Pollutant Emission Standard), SEA (Sudden Environmental Accidents), EVA (Environmental Violations Accidents), EPC (Environmental Petition Cases), IPI-14001 (Is Pass ISO14001), IPI-9001 (Is Pass ISO9001). The value of the corresponding evaluation standard of each enterprise is a Boolean value, that is, it is 1 when the standard is satisfied, and 0 otherwise. For example, if the KPU value of an enterprise is 1, it is a key pollution monitoring enterprise; otherwise, it is not. If the SEA value of an enterprise is 1, it indicates that an environmental accident has occurred in the enterprise recently; otherwise, it indicates that no environmental accident has occurred.

### 2.2. Proposed Method

**Figure 1:**
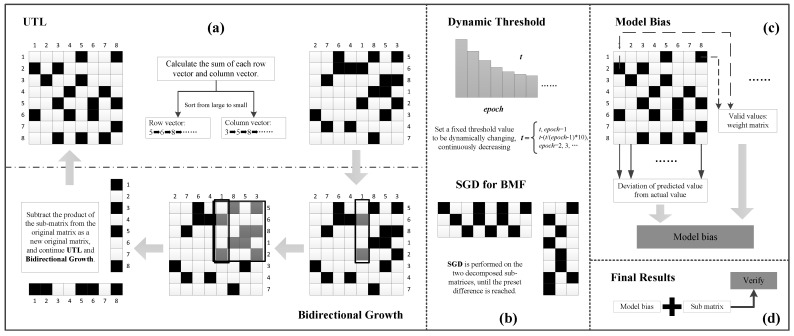
Flow chart of enterprise precise environmental protection behavior supervision method based on BMF. (**a**) Using MEBF to decompose the original matrix; (**b**) A dynamic threshold and an improved SGD are proposed; (**c**) Calculate the model bias; (**d**) Summarize all the methods to get the final prediction results and verify them.

The framework of the method proposed in this paper is shown in Figure 1. Specifically, Figure 1a contains two parts, one is *UTL* (Upper Triangular-Like Matrix), which mainly converts the layout of the original Boolean matrix to form a distribution similar to the upper triangular matrix as much as possible. The other is *Bidirectional Growth*, the core of which is to continuously search for the median column and median row, thereby achieving bidirectional expansion to obtain the decomposed sub-matrix. By continuously updating the original matrix, the sub-matrices are kept updating in parallel to finally obtain the sub-matrices that satisfy the pre-defined conditions. Figure 1b also contains two parts, one is the *Dynamic Threshold*, whose core idea is to optimize the originally fixed coverage threshold into a dynamic threshold, which gradually decreases with the increase in the number of iterations. The second is *SGD for BMF*, which focuses on iterative optimization of the decomposed sub-matrices based on pre-defined conditions to progressively reduce errors and further improve accuracy. Figure 1c is the *Model Bias*, this part reduces the error by determining the weight matrix and incorporating the bias into the prediction matrix. *Final Results* in Figure 1d indicate that the above parts are aggregated to obtain the final results, which are verified by multiple evaluation indicators. Each step of the method will be described in detail in the following subsections, respectively.

#### 2.2.1. Median Expansion for Boolean Factorization

In Section 2.1, the data set selected in this paper is introduced in detail, and it can be found that the elements are all Boolean values. Therefore, the benchmark algorithm selected in this paper is the Median Expansion for Boolean Factorization method (MEBF) [26], which is mainly used for BMF. The core idea of the algorithm is to use the sub-matrix to cover the area with the truest values of the original matrix as much as possible, to update the residual matrix gradually, and to minimize the error between the decomposed submatrix product and the original matrix as much as possible. On the one hand, it is as close as possible to the original matrix elements, and on the other hand, it generates the predicted value, so as to obtain the probability of the enterprise’s environmental protection behavior compliance and achieve precise supervision.

The specific operation of MEBF is represented by Algorithm 1. At the beginning of the calculation phase, the enterprise environmental protection behavior assessment data set is converted into a Boolean matrix *R*, and the coverage threshold *t* is selected. The selection of this value has a great influence on the accuracy of the algorithm. If it is too large, it will cover more noisy information, and if it is too small, more key information will not be fully covered, both of which will reduce the accuracy. *δ*, as the convergence ratio, determines the execution time of the algorithm, and the algorithm will stop running only when the preset error ratio is reached. As the feature dimension of the decomposed sub-matrices, *k* affects the accuracy and time complexity of the algorithm at the same time, it is necessary to choose an appropriate value. The computation phase in Algorithm 1 mainly consists of two steps. One is to *UTL,* the original Boolean matrix. Specifically, the rows and columns in the matrix *R* are numbered in sequence and summed, respectively. The larger the value is, the further up it is placed on the right of the matrix, and the smaller the value, the lower down the left of the matrix. The purpose is to present a distribution similar to the upper triangular matrix and get the matrix R*. The second is to perform bidirectional expansion based on the matrix R*, so that the decomposed submatrices cover the original matrix as much as possible. Specifically, the first sub-matrix model is formed by finding the median column of the matrix and selecting a column vector similar to the median column under the condition of satisfying the coverage threshold, and the indexes covered by the matrix are correspondingly displayed in the sub-matrices *A* and *B*. Then, the sub-matrix model is subtracted from R* to update the matrix R*. By analogy, the above operations are applied to the row vectors until the error of the entire matrix factorization reaches the convergence ratio, or the bidirectional expansion achieves the optimal improvement of the accuracy.


**Algorithm 1:** MEBF

$\mathrm{Input}:\;R\in {\left\{0,\;1\right\}}^{m\times n}$


, t∈(0,1)


, δ


, k∈[1,2,⋯min(m,n)]



$\mathrm{Output}:\;A\in {\left\{0,\;1\right\}}^{m\times k}$


$,\;B\in {\left\{0,\;1\right\}}^{k\times n}$

1:

$\mathrm{Generate}\;0\;\mathrm{matrix}\;A^{m\times k}$


$,\;B^{k\times n}$

2:*UTL* matrix
R:3:    For i←1 to m, calculate the sum of each row vector4:    End for5:    For j←1 to n, calculate the sum of each column vector6:    End for7:    Sort by $UTL,\;R^{*}\leftarrow R$
8:Bidirectional Growth:9:    Start with column:10:      Find the middle column, expand the column (coverage >t), generate submatrix X
11:      If the relative index is overwritten, the corresponding position element becomes 112:     
A←A
, B←B
$,\;R^{*}\leftarrow \left(R^{*}-X\right)$
13:      $\mathrm{If}\;\overset{m}{\underset{i=1}{\sum }}\overset{n}{\underset{j=1}{\sum }}R^{*}÷\overset{m}{\underset{i=1}{\sum }}\overset{n}{\underset{j=1}{\sum }}R<\delta$, cycle steps 10~1214:      End if15:    Start with row:16:Find the middle row, expand the row (coverage
>t), generate submatrix X
17:If the relative index is overwritten, the corresponding position element becomes 118:     
A←A
, B←B
$,\;R^{*}\leftarrow \left(R^{*}-X\right)$
19:      $\mathrm{If}\;\overset{m}{\underset{i=1}{\sum }}\overset{n}{\underset{j=1}{\sum }}R^{*}÷\overset{m}{\underset{i=1}{\sum }}\overset{n}{\underset{j=1}{\sum }}R<\delta$, cycle steps 16~1820:      End if21:return 
A, B



#### 2.2.2. Improvement Measures for MEBF

This section will propose targeted improvement measures based on MEBF. First of all, considering that the [26] has not yet explained how the choice of the coverage threshold *t* is determined, an improper selection will easily have a negative impact on the accuracy of the algorithm, making the choice of precise supervision targets inappropriate, resulting in negative growth in regulatory efficiency. Therefore, this paper proposes a dynamic threshold for the selection of this value, including the selection of the initial threshold and its variation in subsequent experiments. For the selection of the initial threshold, this paper considers that the mean of the median column and median row can be analogous to the overall elements of the matrix. Therefore, the mean of the matrix R* is used as the initial threshold *t* to perform the iterative operation of the algorithm. The dynamic transformation for the value is shown in Equation (1). Among them, the epoch represents the number of iterations of the bidirectional expansion of the algorithm, and t* represents the updated coverage threshold. In short, the coverage threshold for the first iteration is *t*, the coverage threshold for the second iteration is 0.9*t*, the coverage threshold for the third iteration is 0.855*t*, and so on until the algorithm iteration ends.

(1) $$t^{*}=\{\begin{array}{l} t,\;epoch=1\\ t-\frac{t}{\left(epoch-1\right)\;*\;10},\;epoch=2,\;3,\cdots \end{array}$$

Secondly, an SGD method for BMF is proposed to optimize the decomposed submatrices. SGD [27] is an improvement based on traditional batch gradient descent, which does not need to traverse the entire dataset and greatly reduces the time complexity of training. However, it is difficult to apply it to Boolean matrices containing only 0–1 values. When the algorithm is optimal by using this method, the updated value is generally a non-Boolean value, which makes it less meaningful for the optimization application of BMF. That is, it cannot be applied to the enterprise environmental protection behavior assessment data set in this paper. Based on this problem, this paper proposes an SGD algorithm for BMF, as shown in Algorithm 2. Compared with traditional SGD, the proposed method focuses on the updating method of parameters. In this method, the density thresholds *β* of sub-matrix *A* and *γ* of sub-matrix *B* are set before the iteration, and the sub-matrix closest to the preset density is obtained through constant parameter updates. Specifically, firstly, the rows and columns are randomly selected within a limited range, and the parameters of the corresponding positions of the sub-matrix are respectively updated, mainly including two ways. One, if the element in the corresponding position of matrix R* is 1, then there should be element 1 in the corresponding row of sub-matrix *A* and the corresponding column of sub-matrix *B*. At the same time, if the two sub-matrices exceed the limited density threshold, the elements at the corresponding positions are updated to 0. Second, if the element in the corresponding position of the matrix R* is 0, and the corresponding row and column of sub-matrix *A* and sub-matrix *B* are both element 1, there should be at least one element 0, therefore, the randomly selected element is updated to 0. In the continuous update iteration, by reducing the error value until it is smaller than the preset range, the gradient optimization update process ends. This method is integrated into MEBF to obtain MEBF+, which improves the prediction accuracy of enterprise environmental protection behavior compliance.


**Algorithm 2:** SGD for BMF

$\mathrm{Input}:\;R^{*},\;A,\;B,\;\beta ,\;\gamma$



$\mathrm{Output}:\;A^{*}$


$,\;B^{*}$

1:

i=∀ ∈(1,m)


, j=∀ ∈(1,n)


$,\;k,\;k^{\prime },\;k^{\prime \prime }=\forall \;\in \left(1,k\right)$

2:$\mathrm{if}\;R_{i,j}^{*}=1$ do3:  $\mathrm{if}\;A_{i,:}^{*}=0$ do4:    
$A_{i,k}\leftarrow 1$
5:  $\mathrm{if}\;\mathrm{average}\;\left(A_{i,:}^{*}\right)>\beta$ do6:    
$A_{i,k^{\prime }}\leftarrow 0$
7:  $\mathrm{if}\;B_{:,j}^{*}=0$ do8:    
$B_{k,j}\leftarrow 1$
9:  $\mathrm{if}\;\mathrm{average}\;\left(B_{:,j}^{*}\right)>\gamma$ do10:    
$B_{k^{\prime \prime },j}\leftarrow 0$
11:end if12:$\mathrm{if}\;R_{i,j}^{*}=0$ do13:  $\mathrm{if}\;\mathrm{average}\;\left(A_{i,:}^{*}\right)=1$ do14:    
$A_{i,k}\leftarrow 0$
15:  $\mathrm{if}\;\mathrm{average}\;\left(B_{i,:}^{*}\right)=1$ do16:

$B_{k,j}\leftarrow 0$

17:end if18:

$\mathrm{return}\;A^{*}$


$,\;B^{*}$




#### 2.2.3. Model Bias

The algorithm model generally optimizes the results gradually through continuous iteration, but there will always be a certain error with the actual data. Therefore, some scholars propose using the model bias to optimize model results and reduce errors [28]. The core idea is to set weights to individuals with existing data and zero weights to individuals who have not yet generated data, thus, increasing the importance of actual data. For the data in this paper, first, the bias between the environmental protection forecast data of each enterprise and the actual is calculated, as shown in Equation (2). At the same time, since the data involved in this article are all Boolean values, the actual data can be directly incorporated into it as a weight matrix. Then, the effective mean is obtained, that is, the mean value is calculated for the enterprises that generate the actual data. Similarly, applying it to environmental protection behavior indicators, Equation (3) is obtained by analogy with the above-mentioned processing. Finally, the model bias of each enterprise and standard are summarized, as shown in Equation (4), and integrated into the MEBF+ to obtain MEBF++.

(2) $$C_{a,\;1}=\frac{1}{n}*\overset{n}{\underset{b=1}{\sum }}\left(\left(R_{a,b}^{*}-{\left(AB\right)}_{a,b}\right)⊙R_{a,b}^{*}\right)$$

(3) $$D_{1,\;b}=\frac{1}{m}*\overset{m}{\underset{a=1}{\sum }}\left(\left(R_{a,b}^{*}-{\left(AB\right)}_{a,b}\right)⊙R_{a,b}^{*}\right)$$

(4) $$R_{a,b}^{**}=R_{a,b}^{*}+\left(C_{a,\;1}+D_{1,\;b}\right)⊙R_{a,b}^{*}$$

#### 2.2.4. Enterprise Classification

Through the above processing, the predicted value of each enterprise’s next assessment can be obtained. For the value of each standard, this paper considers that the importance of each standard to the enterprise is not exactly the same, thus, the corresponding weight is set, the total score of the enterprise is obtained, then, supervision classification is possible. Specifically, based on the effective role of environmental protection behavior standards on low-carbon goals, the seven enterprise environmental protection behavior standards mentioned above are briefly divided. First, according to the direction of action, it is divided into positive indicators PES, IPI-14001, and IPI-9001, and negative indicators KPU, SEA, EVA, and EPC. Second, according to the degree of importance, it is divided into key indicators KPU, IPI-14001, and IPI-9001, and general indicators PES, SEA, EVA, and EPC. Considering the two classification standards, the specific weight value of each indicator is set by combining the subjective opinion method and the expert scoring method, which are as follows: (1) Key positive indicators: $w_{IPI-14001}=w_{IPI-9001}=1$; (2) Key negative indicators: $w_{KPU}=-1$; (3) General positive indicators: $w_{PES}=0.5$; (4) General negative indicators: $w_{SEA}=w_{EVA}=w_{EPC}=-0.5$. Based on the above-mentioned indicator classification and weight information, and integrating the prediction of enterprise compliance, the total score value of each enterprise is obtained. Based on this, thresholds can be set according to the actual situation to classify enterprises. Different supervision strategies are implemented for different types of enterprises to fully reduce the waste of manpower and resources and improve supervision efficiency.

### 2.3. Evaluating Metrics

To verify the effectiveness of the proposed method, this paper uses four evaluation metrics [29] for verification, namely MAE (Mean Absolute Error), ACC (Accuracy), CR (Cover Rate), and F1 (F1-measure). The specific calculation method is shown in Table 2 and Equations (5)–(8). Since the Precision has an inverse relationship with Recall, that is, an increase in one side will lead to a decrease in the other. Therefore, it is more accurate to use the F1 value for a comprehensive evaluation in this paper. In addition, this paper considers that the evaluation of model prediction accuracy is not completely fair if only using ACC, and it is easy to fall into the dilemma of the accuracy paradox. For example, 99% of the data are negative examples, the model predicts any value will be a negative example and will not predict the result as a positive example. However, its ACC value is as high as 0.99, and its actual effect is very low. Therefore, this paper selects two evaluation metrics, ACC and F1, to evaluate the prediction accuracy of the method. Specifically, (1) MAE can test the degree of error between the predicted value and the actual value. The smaller the value, the more accurate the model prediction is; (2) ACC is the proportion of samples with correct predictions in the total samples. The higher the value, the more accurate the prediction; (3) CR is the ratio of the measurement model to the actual accurate prediction in the population, the larger the value, the more accurate the prediction; (4) F1 is the harmonic average of Precision and Recall. The two are combined to evaluate the prediction results. The higher the value, the better the model performance.

(5) $$\mathrm{MAE}=\frac{1}{n}\overset{n}{\underset{1}{\sum }}\left|r-\hat{r}\right|$$

(6) $$\mathrm{ACC}=\frac{TP+TN}{TP+TN+FP+FN}$$

(7) $$\mathrm{CR}=\frac{TP}{TP+TN+FP+FN}$$

(8) $$F1=\frac{2*\mathrm{Precision}*\mathrm{Recall}}{\mathrm{Precision}+\mathrm{Recall}}=\frac{2*TP}{2*TP+FP+FN}$$

## 3. Results

### 3.1. Superiority Analysis

This paper uses five-fold cross-validation [30] to test the proposed method. The core idea is to randomly divide each data set into five equal parts, and randomly select four equal parts from each experiment as the training set. Based on this part of the data, the model is continuously trained until the error is less than the present value, and the prediction result is obtained. The remaining one is used as the test set to test the prediction results of the model, and the evaluation metrics are used to verify the effectiveness of the proposed method. After prior experiments, the dimension of the decomposed sub-matrices of the model matrix is set to four dimensions, the density threshold *β* of sub-matrix *A* is 0.3, and the density threshold *γ* of sub-matrix *B* is 0.9. During the experiment, each data set was iterated 100 epochs, and then the mean value of each evaluation metric was obtained and analyzed separately.

Table 3 lists the mean values of the evaluation metrics of each data set under different algorithms. It can be found that the performance of the original algorithm MEBF in each evaluation metric is inferior to the MEBF+ and MEBF++ algorithms proposed in this paper. In addition, the performance of each algorithm in datasets of different years is quite different. For example, in the dataset of 2018, the performance of MEBF is the worst compared to the other two datasets, which is due to the sparse distribution of elements in this dataset, which has a large negative impact on the benchmark model. In contrast, the performances of MEBF+ and MEBF++ are still very stable. After longitudinal comparison, it can be found that the improvement is more obvious, which is better than the performance in the other two datasets. At the same time, the targeted improvement of SGD mentioned above focuses on optimizing the factorization and prediction results. When the original prediction results are very poor, its optimization effect is more prominent. Moreover, after the model bias is fused, the model results are further optimized.

**Figure 2:**
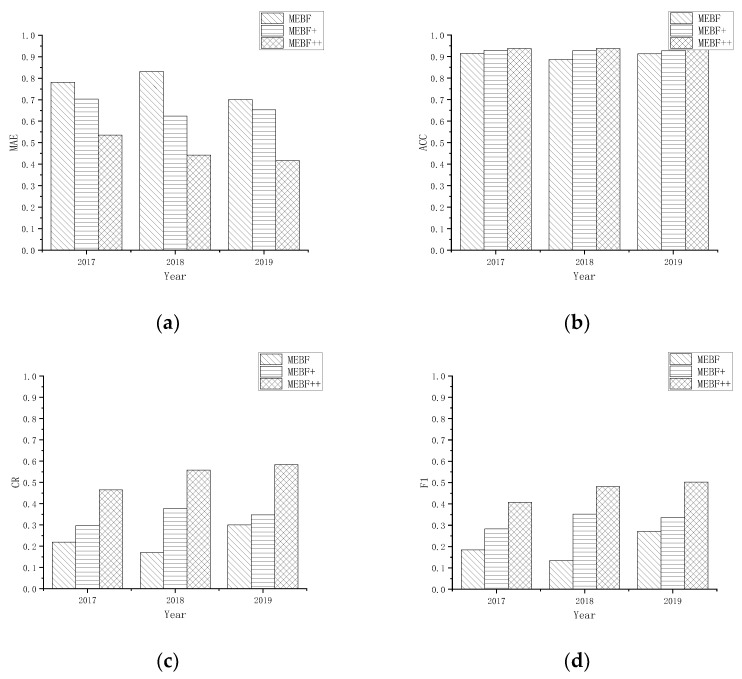
Evaluation and comparison of the running results of each algorithm in different data sets. (**a**) The comparison of the evaluation metric MAE of each algorithm on different data sets; (**b**) The comparison of the evaluation metric ACC of each algorithm on different data sets; (**c**) The comparison of the evaluation metric CR of each algorithm on different data sets; (**d**) The comparison of the evaluation metric F1 of each algorithm on different data sets.

The performance comparison of each algorithm in different datasets is shown in Figure 2. Figure 2a shows the evaluation of the deviation between the predicted results and the real results of each algorithm. It can be found that the original algorithm MEBF added improved SGD to achieve a certain optimization of the original predicted results, and a certain amount of error is reduced in each data set. On this basis, the results are further optimized after incorporating the model bias, which is significantly improved compared to the original results. In Figure 2b, since the benchmark algorithm MEBF has achieved a high degree of accuracy in terms of prediction accuracy, the improvement of the two proposed methods is smaller than the rest of the indicators, but still improved. Figure 2c compares the algorithms based on the CR evaluation dimension. It is found that both MEBF+ and MEBF++ can ensure the optimization of the results and improve the accuracy of the algorithm. Figure 2d uses F1 for comparison; MEBF+ is always better than the benchmark method, the improvement of MEBF++ is greater, and the improvement of the accuracy of prediction results is considerable.

### 3.2. Robustness Analysis

The selection of feature dimensions is very important for the algorithm model. Generally, as the dimension increases, the model accuracy will be higher. However, if the dimension is too high, the time complexity of the model will increase sharply, and at the same time, the phenomenon of over-fitting will occur. Therefore, selecting an appropriate value of *k* can take into account the time complexity and prediction accuracy and obtain satisfactory experimental results. Moreover, the selection of the *k* value is generally artificial and has certain randomness, so a robust analysis of the model for the *k* value is necessary.

**Figure 3:**
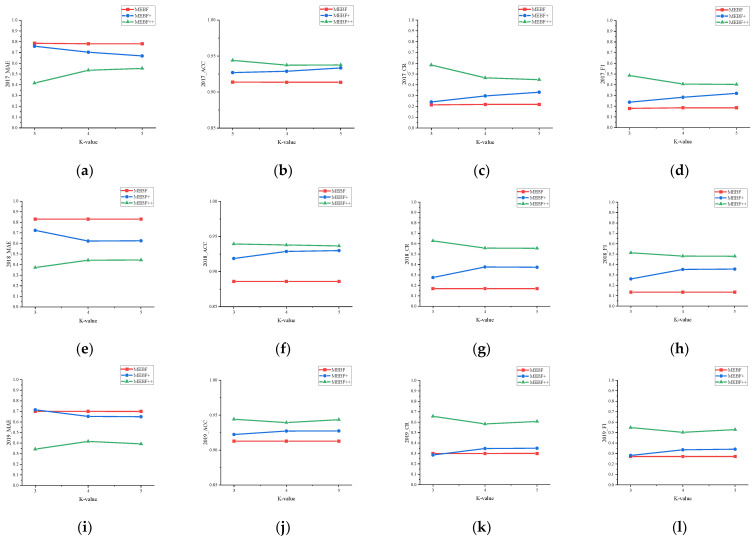
Robustness analysis of algorithms in different datasets. (**a**–**d**) Robustness analysis of the algorithm in dataset 2017; (**e**–**h**) Robustness analysis of the algorithm in dataset 2018; (**i**–**l**) Robustness analysis of the algorithm in dataset 2019.

Figure 3 shows the sensitivity trends of each algorithm in datasets 2017, 2018, and 2019. On the one hand, by horizontally comparing the performance of each evaluation metric in the data set, it is found that MEBF+ is always better than MEBF, and the degree of improvement becomes more and more obvious with the increase in the *k* value. This is because the model advantage is limited when a lower feature dimension is selected, and the higher it is, the improvement is more significant. In addition, compared with MEBF+, the improvement of prediction accuracy is more obvious after incorporating model bias. However, it can be seen that as the value of *k* increases, the improvement decreases and tends to have a stable range. This is because when the value of *k* is small, the accuracy of the model algorithm is low, and the model bias is larger, which can be greatly improved after integration. As the value of *k* increases, the improvement of MEBF++ is smaller than that of MEBF+, but its performance is always the best.

On the other hand, after the longitudinal observation of Figure 3, it is found that no matter how the *k* value is selected, the evaluation metric ACC value of each algorithm remains in a stable state. This is because the performance of the benchmark algorithm MEBF on this metric is already high. On this basis, the improvement of MEBF+ and MEBF++ is limited to ensure that they are not inferior to the former. In addition, the performance of other evaluation metrics MAE, CR, and F1 are all very good, and the performance of the algorithm model will not be degraded with the change in the *k* value. Among them, the change trends of each model algorithm in CR and F1 are very similar, only the values are different. This is because both are evaluations of the prediction accuracy of the model. The difference is that the former is the degree of restoration of the original matrix, and the latter is the comprehensive evaluation of the accuracy of the prediction results.

## 4. Discussion

### 4.1. Effect Analysis

The above has verified the superiority and robustness of the proposed method. In this section, the prediction results of some enterprises will be selected, and the score value of each enterprise will be calculated according to the method in Section 2.2.4. The specific data of some enterprises are shown in Table 4. Based on this, the classification is completed, and then the precise supervision mode is proposed, including narrow precise supervision and broad precise supervision. Both are based on the predicted value of the enterprise’s own indicators and the implementation of appropriate supervision mechanisms. The difference is that the former focuses on the degree of compliance of individual indicators, so that based on each indicator, precise supervision measures are implemented for individual enterprises. The latter focuses on the overall enterprise score, through the classification of enterprises, so as to implement targeted supervision measures for different types of enterprises.

Narrow precision supervision refers to the implementation of targeted supervision mechanisms for different enterprises. This section will take the first two enterprises in Table 4 as examples to introduce the core idea of this type of supervision in detail. In Table 4, the proposed method predicts that the enterprise Shenzhen Cau Technology Co., Ltd. (Shenzhen, China) will meet the two positive indicators of PES and IPI-9001 in the assessment in the coming year and will not violate relevant environmental protection behavior regulations. Therefore, during the period before the next year’s environmental protection behavior assessment, the relevant departments can appropriately reduce the supervision of the company’s environmental protection behavior. By observing the data of Maanshan Iron and Steel Co., Ltd. (Maanshan, China), the second enterprise in Table 4, it is found that this enterprise is more likely to violate relevant environmental protection behavior regulations. On the one hand, although the company complies with pollutant discharge standards, it does not meet two key positive indicators. On the other hand, the company is predicted to be a key pollution monitoring unit and is prone to environmental violations. Through comprehensive consideration, the enterprise will likely have a negative impact on the environment, so the regulatory authorities should focus on supervising the enterprise. In addition, when combined with the actual background of the company, it is found that the above analysis is more in line with the industry types of these two companies. The former is a high-tech enterprise, which can comply more easily with national environmental protection policies, while the latter is an iron and steel complex, which would find it difficult to switch production methods in a short period and comply with environmental protection behavior standards.

**Figure 4:**
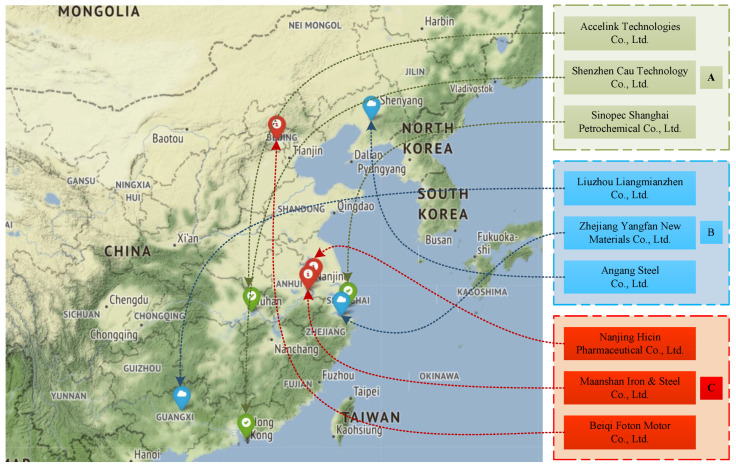
Classification supervision of enterprises.

The core idea of broad precision supervision is to calculate the total score of each enterprise according to the weight of the indicators proposed in this paper. The higher the score, the more likely it will meet the national standards, and on the contrary, the more likely it will violate the national environmental protection behavior regulations. According to the distribution of the overall enterprise score, the corresponding thresholds are set to realize the classification of enterprises, then we can establish a corresponding supervision mechanism for different types of enterprises and adjust the supervision intensity. In order to introduce the operation steps of this strategy in detail, this paper randomly selects nine enterprises that have been processed by the algorithm. The predicted data and total score values of each enterprise are shown in Table 4, and the corresponding thresholds are set accordingly. The top three enterprises are Class A, the bottom three are Class C, and the three middle three are Class B. The specific classification and distribution are shown in Figure 4.

Figure 4 shows the specific classification of enterprises in Table 4. Relevant departments should implement personalized supervision systems for different types of enterprises. In addition, it can be found that the broad precise supervision strategy proposed in this paper is similar to the concept of the positive list system, and both have the ultimate goal of implementing classified supervision. Therefore, this paper will conduct a specific analysis of various types of enterprises based on the above-mentioned enterprise classification, combined with the positive list system. Class A enterprises belong to the group with higher environmental protection behavior standardization among all enterprises, which is reflected in the fact that their environmental protection behavior score ranks at the forefront of the overall enterprises. After observing Table 4, it is found that these enterprises are predicted to meet at least one key positive indicator, and at the same time, the key negative indicators are also predicted to meet with a lower probability. Therefore, such enterprises can act as environmental leaders. The relevant departments can appropriately reduce the supervision of them and give appropriate material and spiritual rewards. The performance of Class C enterprises is opposite to that of Class A enterprises, which are likely to be recorded in the negative list of relevant departments. The main reason is that they hardly meet any of the positive indicators, while they are predicted to meet the key negative indicators, which will have a certain negative effect on the development of a low-carbon society. Therefore, local government departments should increase the punishments for these enterprises, and if necessary, join forces with the public to increase the pressure appropriately, so as to urge enterprises to change their industrial institutions and speed up technological development. Compared with the above two types of enterprises, the Class B enterprises maintain a stable state, which is between compliance and violation of environmental protection policies. This is mainly due to the unbalanced development of such enterprises, some behaviors meet the standards, and some behaviors show violations. For such enterprises, relevant departments can use the above-mentioned narrow precision supervision concept to precisely supervise the violations of various enterprises, and appropriate awards would be given if they meet the standards. In addition, all enterprises in Table 4 comply with the “PES” performance, which means that all enterprises can meet the pollutant emission standards, indicating that China’s departmental supervision has achieved certain results. Enterprises can control basic behaviors, and gradually improve and pursue comprehensive innovation.

### 4.2. Precise Supervision

**Figure 5:**
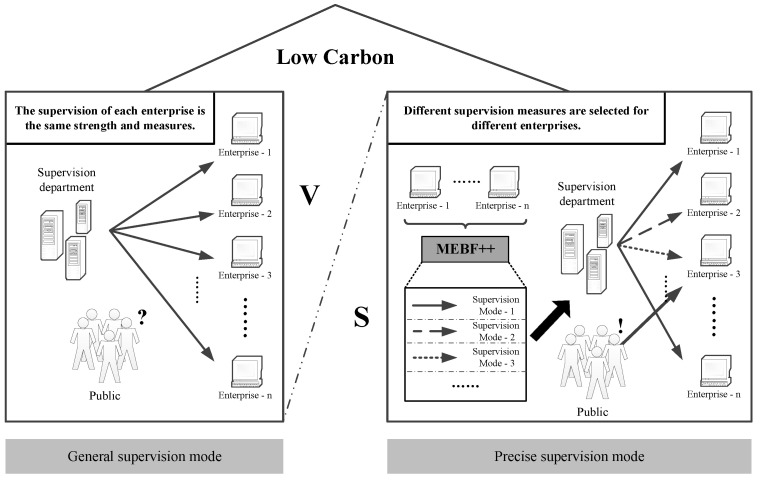
Comparison between general supervision mode and precise supervision mode.

Based on the experimental results above, the proposed precise supervision mode is analyzed in detail. In this section, the similarities and differences between the general supervision mode and the precise supervision mode will be compared. The two-mode diagrams are shown in Figure 5. It is clear from the figure that the similarities between the two are that they operate in a low-carbon background, with the goal of an environmentally friendly society at all times. Moreover, no matter what the supervision mode is, no matter what changes occur, the government is dominant. It should always take the initiative to communicate with other parties, create and mobilize the enthusiasm of the public to participate, and promote the shaping and formation of a benign interaction mechanism. Furthermore, it should always ensure that enterprise environmental protection measures are implemented under supervision and guide all parties to achieve predetermined goals. Both involve the game between the government, enterprises, and the public, striving for the other party to meet their own needs and for the maximum interests of themselves and the group.

In Figure 5, the main differences between the precision supervision mode and the general supervision mode are reflected in the choice of supervision objectives, as well as the supervision strength and strategies. Specifically, in the general supervision mode on the left side of the figure, the supervisory authorities show the same level of supervision for each enterprise, and there is no difference in the corresponding strategies and measures. In addition, the public’s sense of participation is low, and it is difficult to give government supervision a certain amount of help. On the contrary, the precise supervision mode on the right side of the figure fully mobilizes the manpower and resources of all parties and enhances supervision efficiency. Firstly, based on the proposed method, the environmental compliance ratio of each enterprise is predicted in advance. Then, enterprises are classified according to the forecast results, and different types of enterprises will be adopted corresponding regulatory measures. Finally, classified supervision will be completed to maximize the utilization of effective resources. In addition, according to the list of key supervision enterprises announced by the department, which is the negative list. Combined with the positive list, the public can use their limited attention to focus on monitoring some enterprises, focusing on supervising the behavior of potentially polluting enterprises, and assisting the smooth implementation of government supervision.

From the above analysis, we can see that compared with general supervision, the advantages of precise supervision are mainly reflected in two aspects. On the one hand, the general supervision mode is mostly a random selection of targets, lacking a certain theoretical basis, and insufficient accuracy. The precise supervision proposed in this paper can classify enterprises through an algorithmic model to ensure that individualized supervision measures are implemented for various types of enterprises. The government can not only significantly save manpower and material resources, but also cooperate with the public to improve the supervision mechanism to further ensure that enterprise behaviors meet low-carbon standards. On the other hand, by maximizing the use of resources, actively mobilizing the three parties, and fully alleviating the contradictions among all parties, a low-carbon society and the “carbon peaking and carbon neutrality” goal can be more quickly achieved. In addition, the implementation of enterprise environmental protection behavior is conducive to the development of high-tech, and at the same time, it is conducive to the smooth implementation of the national low-carbon policy to ensure the steady development of the national economy and strengthen the sustainable development strategy.

## 5. Conclusions and Policy Implications

By analyzing the existing academic research and practical background, it is found that most scholars focus on proposing new supervision strategies, and there is a lack of research on regulatory target selection. In addition, the relevant departments generally do not treat the supervision of enterprises differently and fail to achieve precise supervision based on the actual situation of the enterprise. The manpower and resource consumption is relatively large and the supervision efficiency is low. This paper conducts relevant research on the above problems, proposes corresponding improvement methods, realizes enterprise classification, and achieves precise supervision. The main conclusions include:

1. This paper proposes targeted improvements for the benchmark method MEBF and obtains MEBF+ and MEBF++. The superiority and robustness of the proposed method are verified based on multiple real datasets;
2. Based on the analysis of the algorithm results, this paper calculates the score of each enterprise combined with the set of the weight of indicator score. Based on this, the classification threshold is determined, and the classification of the enterprises is completed;
3. Comprehensively using the proposed algorithm and analysis results, this paper puts forward narrow precision supervision and broad precision supervision. Based on these strategies, relevant departments can implement a personalized supervision mode for different types of enterprises, realize classified supervision, and improve the supervision mechanism.

Through the above analysis, the supervision departments can adopt the narrow precision supervision mode, broad precision mode, or a combination of both. Based on this, this paper gives some policy implications to relevant government departments [31,32]:

1. For the narrow precision supervision mode, the emphasis is on the implementation of relevant supervision measures for enterprises according to the type of indicators. The seven indicators mentioned in this paper have been divided into four categories in the previous section. First of all, for key positive indicators, if the enterprise meets the standard, appropriate rewards should be given and the intensity of supervision can be reduced. If it does not meet the standard, the punishment should not be too severe, and more attention should be paid to the guidance of their behavior. Secondly, for key negative indicators, departments must strictly control and increase the punishment to avoid the existence of such behaviors of enterprises. Finally, for general positive indicators and general negative indicators, the department can implement corresponding policies based on the actual production situation of the enterprise and conduct partial fine-tuning of the types of indicators under routine supervision, so as to steadily achieve the goals that are in line with environmental protection policies;
2. For the broad precision supervision mode, the emphasis is on the implementation of classified supervision according to the type of enterprise. First of all, according to the classification of enterprises by algorithm, for enterprises with low pollution levels and complying with environmental protection policies, on-site law enforcement inspections should be reduced and spiritual and material awards should be given to boost their social reputation. For enterprises with high environmental risks and weak awareness of abiding by the law, relevant departments should increase their efforts to investigate and deal with environmental violations, increase the frequency of on-site inspections, and urge enterprises to actively improve. For medium-performing enterprises, supervision departments should moderately adjust administrative penalties and reward policies and combine online monitoring with on-site inspections. In addition, the reduction and aggravation in the adjustment process of the supervision system should be moderate to ensure that specific measures are always determined based on the on-site investigation. Additionally, government departments should always reasonably combine law enforcement with law popularization, guide the orderly improvement within enterprises, and jointly achieve low-carbon development.

This paper has made some theoretical and practical contributions to the research in related fields. However, this study still has some deficiencies and limitations, which provide directions for future research. Firstly, the data used in this paper are listed Chinese companies, and unlisted companies have less disclosure of environmental protection behavior information. Therefore, in future work, we can conduct field investigations, cooperate with supervision departments, expand data, and further verify the effectiveness of the method. Secondly, it can be combined with the specific environmental protection behavior indicators of the enterprise and integrated into the algorithm to further improve the accuracy of the method.

## Figures and Tables

**Table 1:** Data sample example.

End Date	Institution ID	KPU	PES	SEA	EVA	EPC	IPI-14001	IPI-9001
31 December 2019	101881	1	1	0	0	0	1	1
	10185	1	0	0	1	0	1	1
	106387	0	1	1	0	0	0	0
	101731	0	1	0	1	0	0	1
	101969	0	1	0	1	0	1	0

**Table 2:** Confusion matrix.

Confusion Matrix		Predictive Value	
		Positive:1	Negative: 0
**True value**	**Positive:1**	Ture Positive (*TP*)	Ture Negative (*FN*)
	**Negative: 0**	False Positive (*FP*)	False Negative (*TN*)

**Table 3:** The mean value of the evaluation metrics of each dataset under different algorithms. Underlined data indicates the value of the benchmark algorithm.

Year	Method	MAE	ACC	CR	F1
2017	MEBF	0.781	0.914	0.219	0.185
	MEBF+	0.699	0.930	0.289	0.299
	**Improve**	10.51%	1.72%	24.22%	38.13%
	MEBF++	0.509	0.939	0.491	0.424
	**Improve**	34.83%	2.74%	124.20%	129.19%
2018	MEBF	0.831	0.886	0.134	0.169
	MEBF+	0.623	0.928	0.351	0.377
	**Improve**	25.03%	4.74%	161.94%	123.08%
	MEBF++	0.442	0.938	0.481	0.558
	**Improve**	46.81%	5.87%	258.96%	230.18%
2019	MEBF	0.700	0.913	0.271	0.300
	MEBF+	0.651	0.929	0.341	0.349
	**Improve**	7.00%	1.75%	25.83%	16.33%
	MEBF++	0.425	0.941	0.505	0.575
	**Improve**	39.29%	3.07%	86.35%	91.67%

**Table 4:** Predicted compliance data of some companies.

	Weight Label	−1	0.5	−0.5	−0.5	−0.5	1	1	Total Score
Institution Name		KPU	PES	SEA	EVA	EPC	IPI-14001	IPI-9001	
Shenzhen Cau Technology Co., Ltd.		0	1	0	0	0	0	1	1.5
Maanshan Iron and Steel Co., Ltd.		1	1	0	1	0	0	0	−1
Nanjing Hicin Pharmaceutical Co., Ltd.		1	1	0	0	0	0	0	−0.5
Beiqi Foton Motor Co., Ltd.		1	1	0	1	0	0	0	−1
Liuzhou Liangmianzhen Co., Ltd.		1	1	0	0	0	0	1	0.5
Zhejiang Yangfan New Materials Co., Ltd.		0	1	0	1	0	0	0	0
Sinopec Shanghai Petrochemical Co., Ltd.		1	1	0	1	0	1	1	1
Accelink Technologies Co., Ltd.		0	1	0	0	0	1	1	2.5
Angang Steel Co., Ltd.		1	1	0	1	0	1	0	0

## Data Availability

The data used to support the findings of this study are available from the corresponding author upon request.
